# On the Applicability of Electrophoresis for Protein Quantification

**DOI:** 10.3390/polym13223971

**Published:** 2021-11-17

**Authors:** Karina Dome, Zoya Akimenko, Aleksey Bychkov, Yuri Kalambet, Oleg Lomovsky

**Affiliations:** 1 Institute of Solid State Chemistry and Mechanochemistry, Siberian Branch, Russian Academy of Sciences, 630128 Novosibirsk, Russia; akzoia56@mail.ru (Z.A.); bychkov.a.l@gmail.com (A.B.); lomov@solid.nsc.ru (O.L.); 2 Department of Business, Novosibirsk State Technical University, 630073 Novosibirsk, Russia; 3 Ampersand Ltd., 123182 Moscow, Russia; kalambet@ampersand.ru

**Keywords:** electrophoresis, protein, mechanical treatment, quantification

## Abstract

Polyacrylamide gel electrophoresis is widely used for studying proteins and protein-containing objects. However, it is employed most frequently as a qualitative method rather than a quantitative one. This paper shows the feasibility of routine digital image acquisition and mathematical processing of electropherograms for protein quantification when using vertical gel electrophoresis and Chrom & Spec software. Both the well-studied model protein molecules (bovine serum albumin) and more complex real-world protein-based products (casein-containing isolate for sports nutrition), which were subjected to mechanical activation in a planetary ball mill to obtain samples characterized by different protein denaturation degrees, were used as study objects. Protein quantification in the mechanically activated samples was carried out. The degree of destruction of individual protein was shown to be higher compared to that of the protein-containing mixture after mechanical treatment for an identical amount of time. The methodological approach used in this study can serve as guidance for other researchers who would like to use electrophoresis for protein quantification both in individual form and in protein mixtures. The findings prove that photographic imaging of gels followed by mathematical data processing can be applied for analyzing the electrophoretic data as an affordable, convenient and quick tool.

## 1. Introduction

Protein chemistry methods are currently used to control, optimize, and elaborate novel technologies in molecular biology, pharmacology, bioengineering, and food technology [1,2,3,4,5]. Such efficient, fast, illustrative, and reproducible methods as high-performance liquid chromatography (HPLC) with different detectors and polymerase chain reaction (PCR) coupled with Sanger sequencing are used for protein quantification [6,7,8,9]. Despite the rapid progress in fast and efficient techniques employed for protein identification and quantification, simpler and more accessible analytical techniques (e.g., the conventional colorimetric measurements) also remain relevant [4,10,11]. Thus, these methods are used for Lowry protein assay in solutions in a reaction with the Folin reagent [11] or Bradford protein assay with Coomassie dye [12].

Protein-containing objects are usually analyzed by 1D and 2D polyacrylamide gel electrophoresis (PAGE), with sodium dodecyl sulfate (SDS) used as a detergent [10,13,14,15,16,17]. Staining with dyes that bind irreversibly to protein molecules but do not form stable bonds with polyacrylamide gel is often employed for protein detection in the gel [18,19,20]. The intensity of stained bands in gel depends on the amount of the applied sample; that is, it is assessed according to the laws of colorimetric measurements: staining intensity is directly proportional to protein content.

Electropherograms are illustrative and informative. However, this technique is most typically used as a qualitative method and quite rarely as a semi-quantitative test (only a visual assessment of band staining intensity is performed). The colorimetric approach (usually the visual one) is also employed in individual cases typically related to molecular biology for measuring the resolution during protein separation in polyacrylamide gel, as well as for protein quantification. In the quantification assay, electrophoretic separation is used together with enzyme-linked immunosorbent assay or western blotting [21,22], which requires respective immune sera against the target proteins.

In recent practical studies, there is demand for protein quantification in complex systems containing numerous impurities of protein and non-protein nature. Previously, polyacrylamide gel electrophoresis was used to determine the depth of hydrolysis of pea seed proteins [23]. The resulting hydrolysate enriched with free amino acids and peptides was used as a component of functional foods. The method combines the qualitative and quantitative assays of a protein mixture by polyacrylamide gel electrophoresis and simultaneous assessment of concentrations of the mixture components. It can also be used to develop special nutrition products containing pea seed proteins [24]. The topic of creating food products from peas is well developed, products containing peas have been mastered by the food industry and are popular [25,26]. In particular, this review notes the positive aspects of the technologies of dry processing of pea seeds.

Electrophoresis in polyacrylamide gel is no less popular in pharmaceuticals. Thus, in order to optimize the procedure for analyzing the drug aprotinin, the time-consuming chemical analysis was replaced by an analysis using HPLC [27]. Meanwhile, as aprotinin derivatives have a protein nature, they can be analyzed by polyacrylamide gel electrophoresis. The target aprotinin and its impurities can be detected by gel electrophoresis as clearly as by chromatography [28]. Thus, the methods of HPLC and electrophoresis in polyacrylamide gel can be interchanged. This approach can also be proposed for monitoring product purification in various bioengineering processes (novel forms of food products [24,29] or novel sorbents for protein purification [30]) and in the development of pharmaceuticals [31].

Therefore, this approach can be employed for manufacturing pharmaceutically important products, such as bovine serum albumin. As a result, simultaneous quantitative and qualitative monitoring of purification of the target product, albumin, will be useful in novel technologies [30].

The patent for an invention of a method for antibody isolation and purification can be mentioned as an example of using this technique for pharmaceutical products [31]. In this and similar studies, it is also convenient and efficient to perform manufacturing process monitoring and simultaneous quantitative assessment of concentrations of immunoglobulin components both during the purification stages and in the target products using PAGE.

The applicability of protein quantification by electrophoresis is currently limited by the following factors [32]. Firstly, there are certain difficulties related to obtaining digital images of the gels. The currently available scanners and densitometers are not common equipment; their resolution is insufficient to work with a densitogram like with a chromatogram. Secondly, the existing software mostly specializes in electrophoresis of nucleic acids and therefore uses a different signal-to-noise ratio [33].

This study makes a methodological attempt to use electrophoresis for protein quantification. The specially designed test bench for digital imaging of gels and optimally selected software allows one to quickly and easily determine the molecular weight distribution of protein molecules in the samples and perform a quantitative assay. This will enable quality control of protein products according to the quantitative contents of fractions of protein molecules and the presence of impurities. The software allows, if necessary, to calculate complex protein samples with diffuse (blurred) protein bands and to exclude unwanted, useless bands on the gel from the calculations. So, the processing of gels allows to obtain more information than other methods: the qualitative and quantitative composition of protein mixtures, as well as their molecular weight distribution.

## 2. Materials and Methods

**Materials.** Bovine serum albumin (BSA; #SLBB7759V, Sigma Aldrich, St. Louis, MO, USA) was used as the model study object. This protein was applied both in its non-modified form and after vigorous mechanical treatment. A protein-containing product, Kultlab Isolate ISO 90% sports nutrition supplement (Kultlab, Novosibirsk, Russia) with 90% casein content, was used as an experimental study object.

The following reagents were also used for electrophoresis analysis in polyacrylamide gel: acrylamide (Sigma Aldrich, St. Louis, MO, USA), SDS (Sigma Aldrich, St. Louis, MO, USA), N,N,N’,N’-tetramethylethylenediamine (Sigma Aldrich, St. Louis, MO, USA), glycine (Sigma Aldrich, St. Louis, MO, USA), tris base (Sigma Aldrich, St. Louis, MO, USA), ammonium persulfate (Sigma Aldrich, St. Louis, MO, USA), dithiothreitol (Sigma Aldrich, St. Louis, MO, USA), glycerol (Sigma Aldrich, St. Louis, MO, USA).

**Mechanical treatment.** In order to obtain samples characterized by various degrees of protein molecule destruction, BSA and the Kultlab sports nutrition supplement predominantly containing casein were subjected to mechanical treatment on an AGO-2 laboratory planetary ball mill (acceleration of the grinding media, 200 m/s^2^). Treatment duration was varied between 5 and 30 min. The weight of the sample loaded into the reaction jars was 5 g per 200 g of the grinding media (steel balls 6 mm in diameter).

**SDS-polyacrylamide gel electrophoresis** was carried out using the Laemmli protocol [20]. Polyacrylamide (Sigma Aldrich, St. Louis, MO, USA) concentration in the stacking and resolving gels was 5% and 13%, respectively. The gel was stained using Coomassie R-250 dye (Thermo Fisher Scientific, Waltham, MA, USA). An unstained protein MW marker (Thermo Fisher Scientific, Waltham, MA, USA) with protein molecular weight ranging between 14.4 and 116 kDa was used as a protein marker.

BSA solution in a lysing buffer (2 mg/mL) for being applied onto the gel lanes was prepared according to the Laemmli protocol [20]. The calibration BSA solutions were prepared by twofold serial dilution. BSA concentration in the calibration solutions ranged from 0.0125 to 0.2 mg/mL. For calibration, the solutions were applied in such a manner that BSA concentration on the polyacrylamide gel lanes was sequentially reduced twofold. The samples of protein mixtures (components of sports nutrition) after mechanochemical activation were prepared using the same procedure. Dilution of sports nutrition samples was selected so that the band intensity lay within the calibration plot.

**Figure 1:**
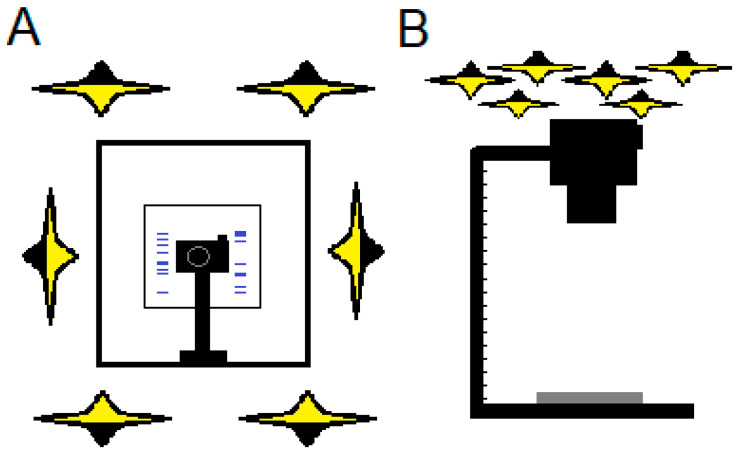
A schematic diagram of the photographic test bench: (**A**) top view; (**B**) side view.

**Protein quantification.** In order to save the electrophoresis results, photos of the gel were taken with a camera (Olympus, Tokyo, Japan) with a 64 MP resolution. The photos were taken on a specially designed test bench with six light sources ensuring uniform illumination of the object (Figure 1).

Taking photos of the gel under these conditions allows one to obtain a densitogram with a resolution being manifold higher than the resolution attainable using scanners for gels. It will be demonstrated below that densitograms can be processed in the same way as chromatograms.

The grey-tone photo images of polyacrylamide gels with stained protein bands were used for protein quantification. Mathematical data processing was performed using the Chrom & Spec software in order to obtain a dependence between protein concentration and band color intensity/peak area [34,35,36]. The results of quantitative measurements were processed and saved using the Chrom & Spec software (Ampersend Ltd., Moscow, Russia).

## 3. Results and Discussion

**Figure 2:**
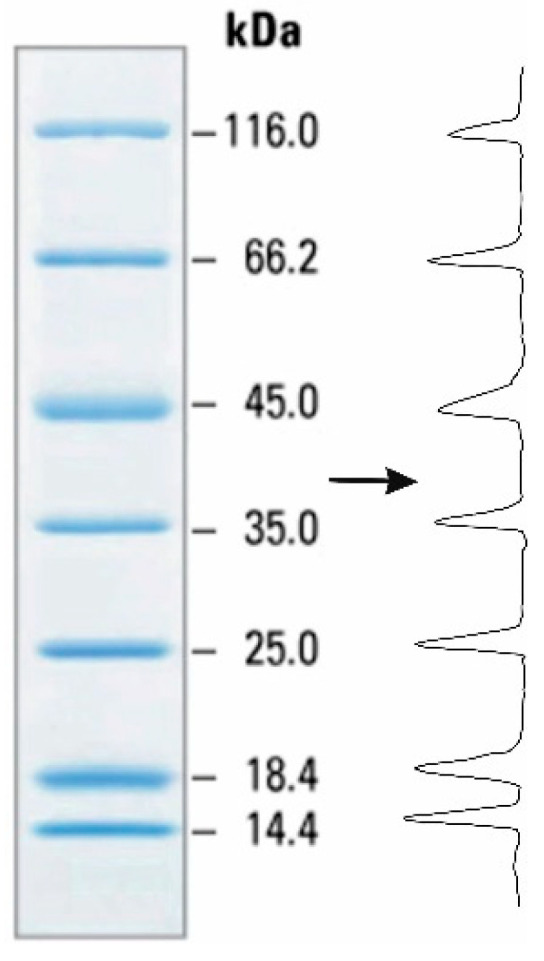
Example of converting the electrophoretic profile of a protein marker into a densitogram using the Chrom & Spec software.

As already mentioned, the electrophoresis results are most often assessed visually, and it is a qualitative assessment. In this study, the results were analyzed using the Chrom & Spec software consisting of two programs: the Planar software for image conversion to densitograms and the Multi Chrom-Planar software performing quantitative processing of densitograms (Figure 2) [34,35]. The area of the resulting chromatographic peaks in the densitogram depends on band color intensity, which corresponds to protein content. The calculations were performed for each peak according to the standard operating procedure of the Chrom & Spec software. A more detailed description of the technical part of the work in the software can be found in Refs. [34,36].

**Figure 3:**
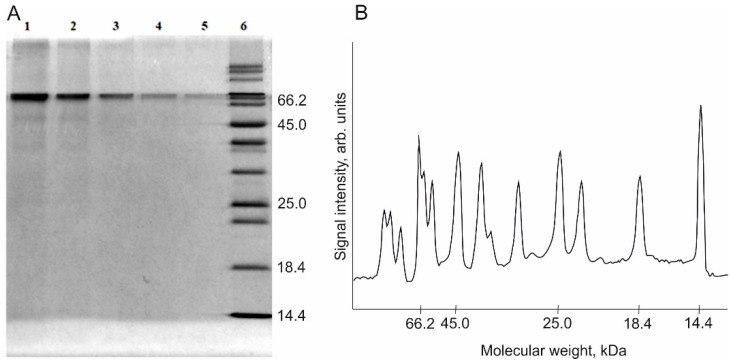
(**A**) An example of the electropherogram of calibration BSA solutions with concentrations 0.2, 0.1, 0.05, 0.025, and 0.0125 mg/mL, respectively (lanes 1–5) and the reference sample with known molecular weights (lane 6). Volume of the applied sample = 10 µL. (**B**) A densitogram of the reference sample.

Figure 3 shows an example of the electropherogram of calibration BSA samples prepared by serial dilution. One can see that band staining intensity in the solutions applied onto the gel varies in accordance with protein content (Figure 3).

The electropherogram was converted to densitograms using the Chrom & Spec software (Figure 3B). As a result of data processing, the area of analytical peaks depending on band color intensity in the gel was measured for the BSA samples with different protein concentrations (lanes 1–5). Table 1 summarizes the results of intensity measurements (peak area in arb. units).

**Figure 4:**
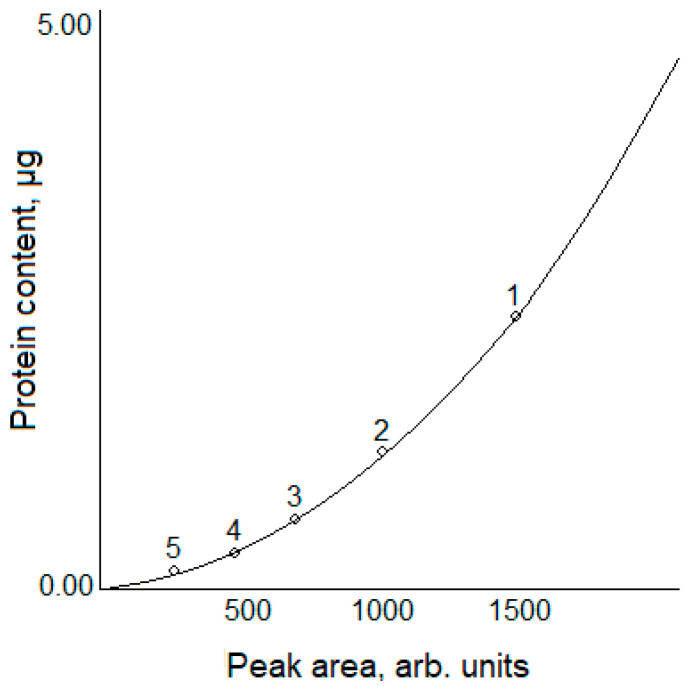
The calibration plot for BSA quantification.

The resulting data were used to build a calibration plot “protein concentration vs. peak area” for BSA (the calibration protein) (Figure 4). A quadratic calibration dependence was obtained:

(1) $$Q=0.1*10^{-5}*S^{2}+2.6*10^{-4}*S$$

where *Q* is the protein content (µg), and *S* is the area of the chromatographic peak on the densitogram. This dependence is standard for planar chromatography or gel electrophoresis [37,38]. The relative deviation was 3.8%. The molecular weight of the analyzed bovine serum albumin (68.53 kDa) was determined using the known molecular weights of the protein marker. The results correlate with the UniProt database values [38,39]. For further studies, the calibration and test samples were applied to the same gel. In this case, all the calculations conducted for the same gel prevent the problems related to the possible non-uniformity of background staining and differences in gel concentration.

**Figure 5:**
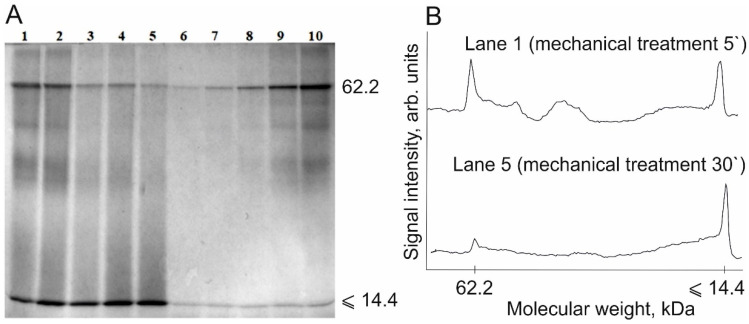
(**A**) Electropherogram of the BSA degradation products after mechanical treatment for 5, 10, 15, 20, and 30 min (lanes 1–5) and the calibration BSA samples with concentration ranging from 0.0125 to 0.2 mg/mL (lanes 6–10); volume of the applied sample = 10 µL. (**B**) Densitograms of the BSA degradation products after mechanical treatment for 5 and 30 min (lanes 1 and 5).

In order to obtain BSA samples characterized by different degradation degrees, they were subjected to mechanical treatment for different times. Sample concentration for electrophoresis analysis was selected so as the intensity of the stained bands lay within the calibration curve plotted for native BSA (shown in the same gel on lanes 6–10) (Figure 5).

**Figure 6:**
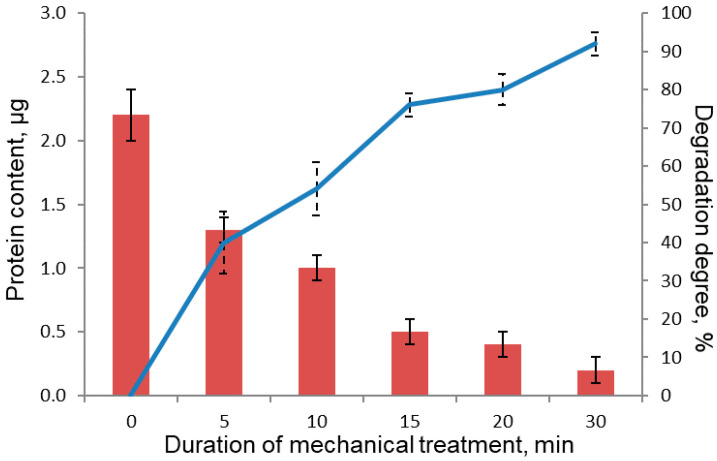
Protein content in the samples of mechanically treated BSA.

The calibration plot was used as the standard of quantitative measurements to calculate the amount of BSA remaining in the sample after mechanical treatment (Figure 6). Figure 6 shows the data on the degree of degradation (α) of protein molecules calculated using the formula:

(2) $$\alpha =\frac{∆S}{S_{0}}=\frac{S_{0}-S_{t}}{S_{0}}*100\%,$$

where ∆*S* is the change in the area of the peak corresponding to the native protein molecule after mechanical treatment (treatment duration t); *S*_t_ is the area of the peak corresponding to the native protein molecule after mechanical treatment (treatment duration t); and *S*_0_ is the area of the peak corresponding to the native protein molecule before mechanical treatment. Protein molecules in BSA subjected to 30-min mechanical treatment were degraded by 92 ± 3%.

**Figure 7:**
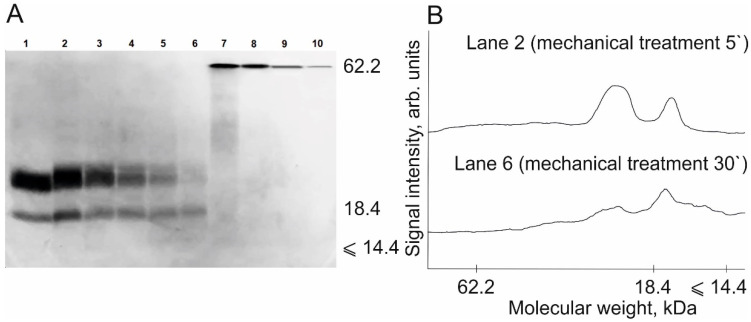
(**A**) Electropherogram of the initial sample of milk protein isolate (lane 1) and the samples of milk protein after mechanical treatment for 30, 20, 15, 10, and 5 min, respectively (lanes 2–6), as well as the calibration BSA samples with concentration ranging from 0.125 to 2.0 mg/mL (lanes 7–10). (**B**) Densitograms of BSA degradation products after mechanical treatment for 5 and 30 min (lanes 2 and 6).

The experiment involving mechanical treatment of milk protein isolate (a sports nutrition mix) and quantitative calculation of casein degradation products during this treatment was conducted in a similar way. Figure 7 shows the electropherogram of the samples of milk protein isolate before and after mechanical treatment for 5, 10, 15, 20, and 30 min. One can see that the destruction of casein protein molecules also takes place during mechanochemical treatment (Figure 7).

**Figure 8:**
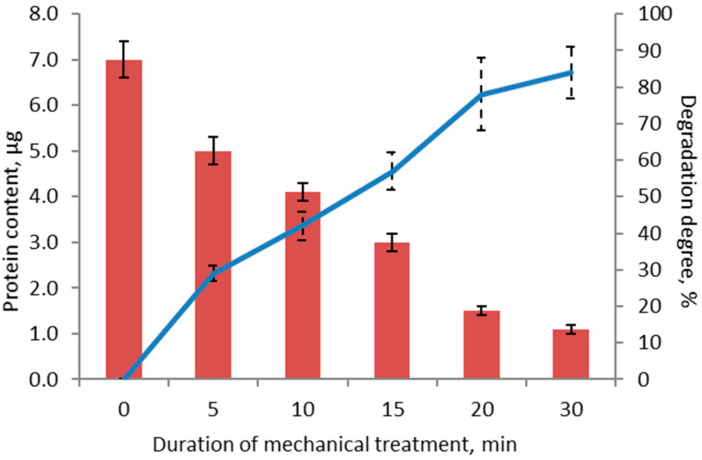
Protein content in milk protein isolate before and after mechanical treatment for 5, 10, 15, 20, and 30 min.

The BSA calibration plot was used to obtain a dependence that allowed one to calculate casein content in the samples (in µg) before and after mechanical treatment for 5, 10, 15, 20, and 30 min. The results are shown in Figure 8. It was demonstrated that the degree of degradation of protein molecules within the sports nutrition product after mechanical treatment for 30 min was 85 ± 2%, being comparable to the data for an individual protein (BSA). The diffusivity (polydispersity) of protein bands as a result of mechanical processing is also noted. This effect is observed in the case of casein proteins, as well as to a large extent for pea proteins in previous research [23]. This circumstance was an important factor for choosing the method of quantitative calculation of protein in the gel, namely, the choice of the Chrom & Spec software.

For the purpose of industrial use of the methodology of quantitative calculation of milk proteins in the gel, a Thermo Fisher Scientific kit has been developed and is used. It includes the following equipment [40]. The procedure allows the quantification of the protein in the gel for various technological tasks, such as the control of protein impurities in dairy products, the regulation of the content of target protein substances with simultaneous control of the distribution of molecular weight. However, for unique research work, such a kit may be inconvenient, expensive, and unavailable. An application for smartphones has been developed for the express processing of gels, which allows the determination of the molecular weights of fragments of deoxyribonucleic acid or proteins with high accuracy [41]. This approach is very interesting because its use does not require specific equipment. However, this application does not allow quantifying analytes. The method proposed in this article allows you to quickly obtain information about the molecular mass distribution of molecules in the gel, as well as the quantitative and qualitative composition. Also, the Chrom & Spec software allows the use of not only standard operating procedures but to choose the appropriate mathematical processing independently, depending on the task set by the researchers. This requires enough standard equipment such as a personal computer with the necessary software and a camera (it is also possible to use a scanner or smartphone), so this approach is available to a large number of researchers working with electrophoresis.

Hence, it has been shown that polyacrylamide gel electrophoresis coupled with simultaneous recording photographic images of the gels and mathematical data processing using the Chrom & Spec software allows one to measure protein content in the test sample directly in polyacrylamide gel (identically to the known colorimetric methods for protein quantification). This technique has made it possible to estimate the degree of protein degradation for the model BSA protein and casein (a component of sports nutrition products). The procedure allows protein quantification for various applied problems such as performing control over protein impurities or regulating the content of target protein substances with simultaneous control over the molecular weight distribution.

## 4. Conclusions

In this work, we used photographic visualization of gels followed by mathematical data processing using Chrom & Spec software to quantify the intensity of protein bands in polyacrylamide gel. The results obtained proved that this algorithm can be used to process electrophoretic data and obtain accurate data regarding the quantitative analysis of proteins using this method. The relative inaccuracy of the method was estimated using calibration solutions of BSA. To make the protein quantification more accurate, it was proposed to use calibration solutions together with test samples on a single gel so that all variable factors could be taken into account during the analysis and recording of the results.

The possibilities of the presented method were tested on BSA and casein in the composition of a sports nutrition product, which were subjected to mechanical processing. The proposed method was used to obtain data on the relationship between the degree of degradation of protein molecules and the duration of mechanical processing. Mechanical treatment of BSA for 30 min resulted in degradation of protein molecules by 92 ± 3%, while protein molecules in a sports nutrition product were degraded by 85 ± 2%. The degree of destruction of an individual protein was higher compared to the degree of destruction of a protein-containing mixture after mechanical treatment for an identical period of time.

The methodological approach used in this study can serve as a guide for other researchers who would like to use electrophoresis to quantify protein both in individual form and in protein mixtures.

## Figures and Tables

**Table 1:** The resulting data for plotting the calibration plot.

Lane Number	Protein Content in the Sample, µg	Peak Area, Arb. Units
1	2.0	1288 ± 49
2	1.0	934 ± 35
3	0.5	763 ± 29
4	0.25	525 ± 20
5	0.125	348 ± 13

## Data Availability

The data presented in this study are available on request from the corresponding author.
